# Translation research: from accurate diagnosis to appropriate treatment

**DOI:** 10.1186/1479-5876-2-35

**Published:** 2004-10-21

**Authors:** Craig P Webb, Harvey I Pass

**Affiliations:** 1 Principal Investigator, Laboratory of Tumor Metastasis and Angiogenesis and Director, The Multiple Myeloma Research Laboratory, 333 Bostwick Avenue, Van Andel Research Institute, Grand Rapids, Michigan 49503, United States; 2 Thoracic Oncology, Karmanos Cancer Institute/Wayne State University, Harper University Hospital, 3990 John R, Suite 2102, Detroit, Michigan 48201, United States

## Abstract

This review article focuses on the various aspects of translational research, where research on human subjects can ultimately enhance the diagnosis and treatment of future patients. While we will use specific examples relating to the asbestos related cancer mesothelioma, it should be stressed that the general approach outlined throughout this review is readily applicable to other diseases with an underlying molecular basis. Through the integration of molecular-based technologies, systematic tissue procurement and medical informatics, we now have the ability to identify clinically applicable "genotype"-"phenotype" associations across cohorts of patients that can rapidly be translated into useful diagnostic and treatment strategies. This review will touch on the various steps in the translational pipeline, and highlight some of the most essential elements as well as possible roadblocks that can impact success of the program. Critical issues with regard to Institutional Review Board (IRB) and Health Insurance Portability and Accountability Act (HIPAA) compliance, data standardization, sample procurement, quality control (QC), quality assurance (QA), data analysis, preclinical models and clinical trials are addressed. The various facets of the translational pipeline have been incorporated into a fully integrated computational system, appropriately named Dx2Tx. This system readily allows for the identification of new diagnostic tests, the discovery of biomarkers and drugable targets, and prediction of optimal treatments based upon the underlying molecular basis of the disease.

## The Ultimate Goal

**Figure 1:**
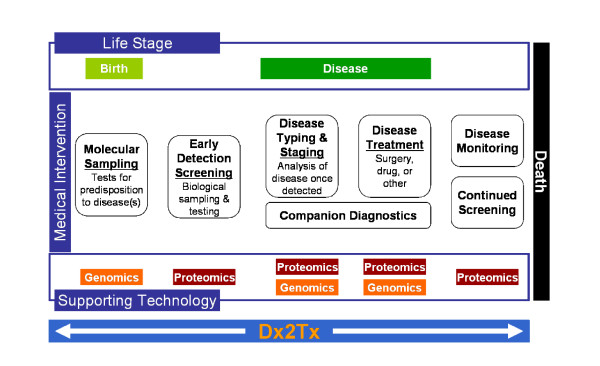
The goal of our translational research effort – From Diagnosis to Treatment (Dx2Tx). We have developed a systems approach to track pertinent clinical events within the lifespan of an individual subject. In the future, it may be possible to predict the risk of a clinical event in advance, accurately diagnose the event in its earliest manifestations, and treat based upon the underlying molecular/clinical traits. We believe the integration of medical informatics with cutting edge molecular technologies such as genomics and proteomics will expedite this transition to molecular-based medicine.

Our systematic approach to translational medicine has been designed to achieve a vision shared by numerous "ambitious" investigators who are focused on applying bench-side discoveries to the clinical setting (Figure 1). We share a vision of future medicine, in which it will be possible to predict the likelihood (risk) of a clinical event during the course of an individual's lifetime, accurately diagnose the event in its earliest manifestation, and treat accordingly based upon the diagnosis. We believe that this will become reality through the combination of medical informatics and the multiplex "omics" technologies (that we broadly characterize as genomics and proteomics throughout this review) now at our disposal. Clearly, there are certain bioethical, political and fiscal roadblocks which need to be considered as we progress towards this goal, not limited to patient privacy, regulatory issues, health care reimbursement and ownership of intellectual property. In this review however, we will focus on the science and logistics relating to implementation of a successful translational research program.

**Figure 2:**
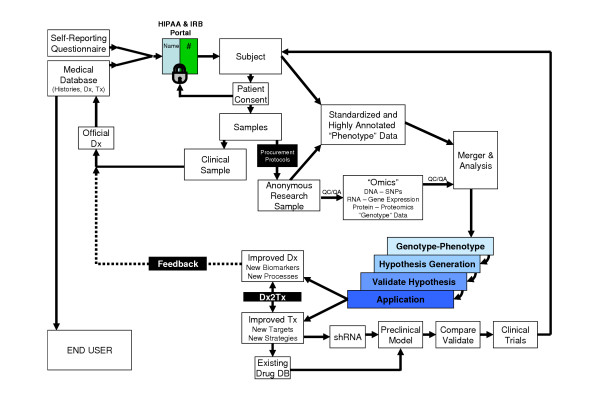
Systematic overview of translational research. Commencing with human research subjects, we can transition through specimen and data collection, data analysis, preclinical models and ultimately clinical trails. The various facets of this pipeline will be discussed throughout this review.

**Figure 3:**
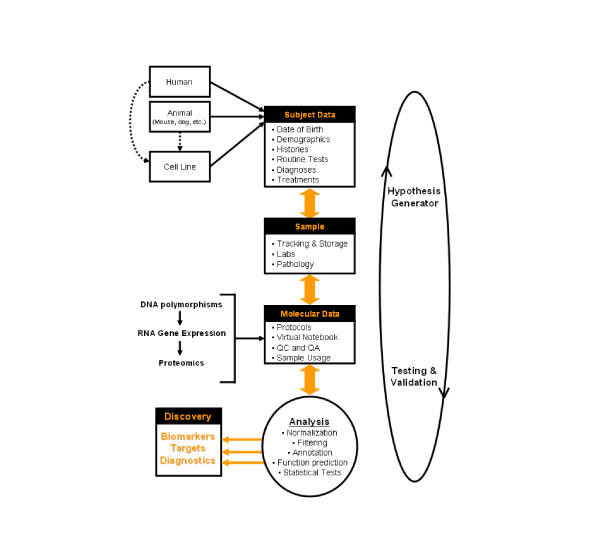
The Dx2Tx integrated solution stores and analyzes clinical (and preclinical), experimental and molecular data from a variety of disparate sources. For more information see

While a degree of project specificity must be incorporated into the design of any research effort, many of the components of translational research are shared across apparently disparate disease areas (Figure 2). Although many facets of a research proposal (i.e. attaining funding and IRB approval) require specification of the particular hypothesis under evaluation, we also make every effort to collect samples (such as blood, urine and tissue) together with as much clinical data (both historical and longitudinal) as possible while maintaining patient confidentiality, privacy and safety at the forefront. In addition to testing the pre-conceived clinical hypothesis (for example, does an event have an underlying molecular basis), this approach readily allows for the discovery of additional genotype-phenotype patterns (hypotheses) that can be subsequently cross-validated in additional subjects and samples (i.e. this event does have an underlying molecular basis). This somewhat blinded yet methodical approach clearly requires a database and supporting analytical software that are intricately linked, such that analytical results can become a new query against the database content (Figure 3). While these aspects of the Dx2Tx system will be discussed in more detail throughout this review, it is essential to stress the importance of collecting and archiving standardized clinical data throughout the course of a patient's lifespan. It is difficult to identify statistically significant correlations within non-standardized datasets. Well documented clinical data is equally as important as clinical samples, a fact often overlooked in many research studies reported to date.

Effective translational research requires a multi-disciplinary and team approach. Our initial concept meetings include HIPAA/IRB advisors, research nurses, surgeons (and/or other health care providers such as oncologists), pathologists and members of diagnostic service labs, information technologists (both biological and medical disciplines) and statisticians in addition to the principal investigators. Each member of the translational research team is critical to the overall productivity and success of the pipeline. In acknowledgement of the effectiveness of this approach, we would advise other establishments to involve key personnel across the various disciplines early. We also believe that smaller dedicated teams can often be more efficient and less bureaucratic than oversized consortium.

## IRB, HIPAA, Scheduling and Consenting

The development of research protocols on human subjects that involve specimen and/or data collection must be approved by the investigational review boards (IRB). The IRB governs patient safety and risk for the hospitals and/or universities/institutions. In accordance with federal regulations, one of the requirements states that each human subject must be thoroughly informed about the research to be undertaken on their sample and/or data during a consenting process. The specificity of the consent should be such that it outlines the research protocol, study procedures and risks and benefits and meets the federal, state and local requirements, so that the subject can make an informed decision regarding participation in the study. In retrospect, a well informed patient can help facilitate the procurement of clinical data and samples in an efficient and longitudinal manner.

While the typical testing of a hypothesis requires the collection of only a subset of the available clinical data (for example tumor stage, survival time), the emerging field of medical informatics and electronic charts allows for the potential collection of vast quantities of standardized clinical data that potentially harbors invaluable information with regard to additional "traits" or "phenotypes". As such, we currently collect upward of 9000 potential data fields per patient within our specific IRB-approved protocols, all of which can be populated during the normal course of patient care and stored within either electronic or paper records for each subject. In addition, each patient receives a standardized self-reporting questionnaire addressing histories (such as habits, family history, medication history, etc). Any protected fields are restricted through a security portal, which can be considered a suitable clinical gatekeeper (such as research nurse or electronic portal) (Figure 2). This security portal allows the flow of clinical data to the laboratory researcher as defined in the IRB-approved protocol, and also links each subject/sample with an anonymous ID. Patient data that is de-identified in this fashion is not subject to HIPAA regulations. It should be noted that some protected health information (such as zip code) may represent valuable information to the research team (for example when addressing potential local or regional causes of disease incidence). Patients are informed and provide written authorization for the inclusion of such potential identifiers during the consenting process; these are then collected and archived in secure locations. For example, we routinely include current home and work 5-digit ZIP codes and date of birth in our IRB-approved protocols. The latter is used to normalize patients based upon age of each clinical event, where date of birth represents time zero in a patient's life span. As such, every recorded event in a patient's life is tagged to a date and time, such that events can be readily interrelated. For more information on HIPPA and sharing of PHI for research purposes, see .

In addition to IRB and HIPAA issues, patient recruitment can also be a major hurdle. The teamwork approach is critical here, together with patient/physician outreach and screening efforts as necessary. Our experience is the vast majority of patients are enthusiastic about inclusion in research studies so long as they are informed of the opportunity, particularly since we are careful to follow standard-of-care. For the most part, we are finding a willingness to participate for the hope of betterment for the whole, whether it is potentially helping their own families or others with their disease. At this level, the physician is the central figure and is uniquely positioned to introduce the protocol and support patient participation in the study. For studies where low participation is expected (predominantly due to low incidence), we typically include an IRB-approved public educational effort (for both physicians and potential patients) that maximizes recruitment. Our clinical research nurses work closely with the physician(s) and their office scheduling staff for notification of potential candidates in advance wherever possible. This team approach with the physician advocating the effort allows the greatest accrual particularly when there are several geographic sites for meeting potential protocol volunteers. On site consenting of high risk or suspected individuals within the outpatient clinic and hospital settings where these patients are typically evaluated should to be considered to enhance accrual. For patients with known disease, the best environment for accrual occurs in a multidisciplinary setting in which the key physician works closely with the medical oncologist, radiation oncologist, nurses, physician assistants, residents and research staff. This is particularly important for longitudinal sampling of patients on treatment protocols to minimize the loss of follow-up data. The logistics of a coordinated approach may differ between a university medical school setting and a community based practice due to the competing obligations of a busy private practice. The multidisciplinary approach is optimal in a single setting in which patients can be seen by the various disciplines, thereby, reducing the extent of patient relocation between specialty offices and allowing for centralized specimen and data collection. Nonetheless, in community-based practice, we have introduced standardized mechanisms allowing us to consent patients and collect clinical samples and data from multiple sites.

## Clinical Data Acquisition

There are now abundant examples where clinical researchers are using the revolutionary "omics" technologies to demonstrate a clear association between the molecular make-up ("genotype") of clinical samples and well-defined clinical characteristics ("phenotype"). Near-complete genotypes can now be obtained through multiplex technologies such as sequencing, mutational screening (such as single nucleotide polymorphism (SNP) analysis) and gene/protein expression profiling. The absence of well annotated clinical information (such as medications, response to treatments, histories, environmental exposures, toxicities, etc), however, is evident from most studies reported to date. Rather than taking the reductionist approach at the time of data collection, our group instead chooses to gather all information relating to an individual's medical history as well as follow-up data as it becomes available and as approved by the IRB. It is relatively trivial to reduce the clinical data pool retrospectively during analysis as deemed necessary. Our Dx2Tx system date/time stamps individual events for a given subject/sample, such that multiple events can be temporally associated. The first step is to gather the information in its rawest form from a standardized source. This could be as complicated as a centralized medical informatics database (such as the Oracle-based clinical informatics system used by Spectrum Health Hospitals (Grand Rapids, Michigan) housing rich longitudinal clinical data that can be expressed in Health Level 7 standards for data portability), or it could be as uncomplicated as a locally maintained excel spreadsheet or access database. Regardless of the source, we attempt to collect as much information regarding a subject's history, diagnosis, treatment, and response assessment as possible. As highlighted above, the critical elements are reliability of the source data and standardization. While we do perform statistical analysis on parsed text from open string comments, we attempt to force objective measurements (such as integers, floating numbers, text pull downs, binary data, etc) wherever feasible. In the absence of a centralized clinical database utilizing standardized clinical nomenclature and data, the responsibility falls on the clinical members of the team to interpret clinical data within isolated databases and/or paper charts. Clearly, the navigation from paper to electronic medical records, and the generation of middleware that can link disparate databases, will greatly alleviate this burden that rapidly becomes a major rate-limiting step in the translational pipeline. Coupled with voice recognition and electronic data recording with inbuilt QC check-points, standardized digital data entry should become the norm in the not so distant future.

Clinical data permitted under protocol should be, where possible, prospectively accumulated. The accuracy of the data, particularly those parameters such as the pathologic characteristics, clinical staging, dates of intervention, dates of intermediate endpoints (such as disease progression or death), must be unquestionable in order to identify clinical and molecular correlations among diverse datasets. The accuracy of these data will depend to a large extent upon the frequency of follow-up for parameters such as disease progression, and the degree to which the patients' status is investigated longitudinally. Patients on clinical trials, where the procedures and follow-up are specified as part of the protocol, will have the most robust and standardized data since interval radiographic and physical examinations and/or other clinical procedures and methods of data collection must be adhered to in order to avoid protocol violation. Moreover, the approach of the physician(s) who follows the patient and the availability of an existing mechanism that readily allows the retrieval of prospective data (i.e. a database) will greatly influence the accuracy of the data. Within oncology, staging issues are some of the more difficult to resolve, especially if inadequate staging is performed in the operating room (OR) or if one must rely entirely on clinical or radiographic data. For the majority of organ systems, the oncological staging system is specified in the AJCC Staging Handbook, but it is the responsibility of the investigator to know whether the pathologic and intraoperative details necessary for accurate staging were adequately performed. These include but are not limited to accurate size measurement of the primary tumor, careful intraoperative description of abutment or invasion of nearby structures, verification of negative margins, and extent of lymph node involvement. The patients used in the examples below were all part of either Phase II or Phase III mesothelioma protocols, with computerized tomographic surveillance after surgery every 3–4 months until death. As such, the accuracy of the clinical data was optimized. One surgeon performed the operations, obtained aggressive intraoperative staging in all cases, and took the responsibility for collecting samples and data from all participants in the study. This approach led to the development of a consistent standard of care philosophy for the surgical management of mesothelioma.

Our Dx2Tx system performs detailed statistical analysis on both clinical and molecular data, thus leaving little room for error when it comes to standardized data collection and entry. Physicians are uniquely positioned to organize the various aspects of data collection. This endeavor begins with the design of a clinical protocol for the procurement of specimens and the collection of data from a patient population. In addition to identifying the experimental group of patients (i.e. those with a condition or trait of interest), it is important to ensure the inclusion of the proper control populations wherever possible. With respect to our mesothelioma protocols, the correct controls for early diagnostic strategies include age-matched individuals exposed to similar doses of asbestos in order to compare with equivalent individuals diagnosed with the established disease. The recruitment of these control subjects ideally begins in parallel with the recruitment of the experimental group. In the absence of the correct control group, the investigator must identify cohorts of individuals within existing cooperative group mechanisms, SPORES, or the Early Detection Research Network (EDRN). In order to circumvent this problem, the Karmanos Cancer Institute has established a National Center for Vermiculite and Asbestos Related Cancers through a cooperative clinical trial with the Center for Environmental Medicine in Southeast Michigan. This center, under informed consent, evaluates individuals who have been exposed to asbestos in the workplace or at home, and depending on history and pulmonary function data, these individuals undergo computerized tomographic scanning to establish a baseline radiographic evaluation of asbestos exposure and risk. These individuals also give written permission for periodic sample collection including blood and urine for studies of marker assessment for asbestos related malignancies.

A major focus of our research is to identify the molecular causes of differential therapeutic response across patient populations (pharmacogenetics). It is generally appreciated that patients and their disease show significant variations in response to a given treatment [1]. It is therefore critical to develop standardized approaches for routinely monitoring adverse responses (for example using common toxicity criteria) as well as disease response (for example using the Response Evaluation Criteria in Solid Tumors, or RECIST criteria). While various reporting schema are in place under specific clinical protocols, we are working on a more systematic approach; the collection of raw clinical data that can be compiled to assess clinical response and toxicity. Accordingly, if the raw data is collected, the response of all patients undergoing defined treatments can be assessed in a longitudinal fashion. The compiled response and toxicity data can then be analyzed retrospectively with other clinical and molecular attributes as outlined below.

## Specimen Collection and Archiving

We have introduced standard procurement procedures for various physiological samples including tissue, urine and blood, all of which are now routinely collected under our IRB-approved protocols. These have been optimized for both ease and reliability of clinical collection as well as maintaining the integrity of the sample for subsequent histopathological and molecular analysis. DNA from peripheral blood mononuclear lymphocytes (PBML) obtained from whole blood can be screened by SNP analysis, to identify possible genetic markers of a clinical event [2-4]. Once validated, these SNP markers could serve as a genetic test to predict the risk of the event in prospective subjects. Plasma or serum (we prefer non-clotted plasma) and urine can be screened for proteomic markers of a clinical incident, and used in future screening for diagnostic purposes [5]. Per the approved protocol, tissue can be subjected to a variety of DNA/RNA/Protein technologies, and important diagnostic and therapeutic insight gained at the molecular level [6]. It should be noted, that wherever possible, adjacent uninvolved "normal" tissue free of disease should also be collected for comparison with the corresponding diseased tissue. The example in this review will focus on some published works [7] identifying diagnostic and prognostic biomarkers in the tumor tissue of patients with mesothelioma utilizing Affymetrix GeneChips for assessing gene expression within the tissue. In this case, we collected normal mesothelium of the peritoneum or the pleura in conjunction with mesothelioma tumor tissue.

The precise flow of the clinical sample from the patient to the researcher will depend largely on the disease in question and pre-existing departmental procedures. For example, in a study of pancreatic cancer, urine is provided by the patient prior to the procedure, since we have found that intra-procedural catheterization can result in blood contamination that effects subsequent proteomic analysis. Blood is drawn from an IV access for collection of plasma and PBML, while surgically resected tissue is snap frozen on site (see below). However, a multiple myeloma research protocol requires the collection of bone marrow aspirates from both inpatient and outpatient clinics, which are placed into tissue culture media and rapidly transported on ice to the Flow Cytometry Molecular Diagnostic Laboratory for immunophenotyping and cytometric sorting of plasma cells. The sorted fraction is then archived in freezing media for subsequent DNA, RNA and/or protein analysis. Irrespective of procedure, it is important to maintain a log book that tracks the flow of a sample from the subject to freeze. Procurement time is particularly important for RNA and some protein analysis, since biomolecular degradation can be a significant factor.

Prior to collection, our procurement team (lab personnel and research nurses) pre-label collection tubes and containers with anonymous identifier tags that are freezer safe. Several kits that include equipment for blood draws, urine collection and tissue procurement are preassembled and made available to the research staff to ensure rapid response for obtaining specimens. We have an on-call list of several members for the research team who can reach the pick-up point within 30 minutes of receiving a collection call. Through a coordinated multidisciplinary approach, consenting and scheduling is typically obtained well in advance in either the outpatient or inpatient setting.

At the Karmanos Institute, we have found that the easiest way to insure proper specimen harvest from patients with solid tumors is for the surgeon to divide the specimen directly in the OR and supervise its collection and distribution to the laboratory for archiving. However at the Van Andel Research Institute, we require oversight from the Hospital Department of Pathology to release surgically resected samples within the OR to the research team. The critical issue is to ensure suitable material is harvested in the correct fashion to allow accurate histopathological diagnosis. In no way should the research effort impede this quality-of-care. As a safety margin, we hold all frozen tissue for a period of time until an official diagnosis has been reported. In addition, wherever possible we embed samples in OCT freezing media, and generate hematoxylin/eosin (H/E)-stained histopathology slides for each research sample. This not only provides high quality histopathology slides for possible diagnostic back-up, but also allows us to correlate our molecular findings with the histology of the same working sample. In addition to entering pertinent data regarding the official diagnostic pathology report, we have also developed standardized report templates within Dx2Tx that accompany each research sample, in which the pathological image is associated with the corresponding data addressing critical variables such as relative amount of each cell type, stage, morphology etc. As with all data entered into Dx2Tx, these metadata are directly amenable to subsequent statistical analysis (see below).

At the time of resection, solid tissue samples are immediately frozen in either liquid nitrogen or, for sites with no supply, a dry ice-chilled isopentane bath. Samples are transported in aluminum foil, and stored at -80°C until further processing. It should also be pointed out that many of our protocols involve the collection of tissue biopsies in addition to surgical resections. Since it is physically difficult to split a biopsy (although this is done for example with bone marrow core biopsies), we typically acquire additional samples beyond what are required for accurate pathological diagnosis. If these additional samples are required by the research team, it is important to disclose the supplementary procedures to the patient in the informed consent. While obviously smaller in size compared to surgical resections, we procure these specimens as fresh tissue as described above. It is important to note, some of our molecular technologies allow for the use of as few as 500 cells for complete multiplex analysis. Since biopsy material will doubtless be the predominant source of tissue for future molecular-based diagnostics, it is important to develop protocols to address smaller sample size and possible mis-sampling issues. In addition, as a result of not restricting samples to excess surgical specimens, biopsies can rapidly increase sample accrual from larger cohorts of patients.

Urine and blood samples are transported on wet ice to the research labs, while frozen tissue is transferred on dry ice. Upon arrival in the laboratory, urine is typically divided into suitably sized aliquots and frozen at -80°C. Blood is fractionated into plasma or serum (again aliquoted and frozen), while PBML are isolated by centrifugation and Ficoll/Hipaque density gradient centrifugation and cryogenically frozen. Fresh frozen tissue specimens are blocked in OCT if possible and stored at -80°C until further use. All specimens are associated with the donating subject within Dx2Tx, and archived in a simple grid system within dedicated and alarmed -80°C freezers. The location and use of each aliquot is tracked within Dx2Tx to readily allow for subsequent retrieval.

## Sample Procurement – The "Omics"

The tissue collection protocols outlined above should be evaluated for compatibility for the molecular analysis to be performed. We typically perform SNP analysis on DNA isolated from the PBML fraction of whole blood, gene expression (mRNA) analysis on tissue, and proteomic analysis of blood plasma/serum, urine and tissue. The detailed discussion of each of these molecular protocols is beyond the scope of this review (see [6] for a good starting point of reference). There are important differences between the various platforms available that can result in some disparities in the results generated [8-10]. As such, it is essential to document and ideally database protocols, deviations from protocols (version control), and in general, all experimental variables that could potentially confound the results (for example see Minimum Information About a Microarray Experiment ). We enter these variables into Dx2Tx in association with the corresponding experimental procedure such that they can be analyzed together with the derived molecular and clinical data. In this fashion, important QC and QA parameters can be interrelated with molecular and clinical data. Experiments that do not meet certain QC/QA criteria can be excluded from the analysis if the data suggest that the variable(s) is a major confounder. In this fashion, Dx2Tx serves as an electronic notebook, the entries into which can be routinely recorded and statistically analyzed. This feature will be particularly important as potential applications advance towards clinical utility, for example through ensuring compliance with the FDA and accrediting agencies such as JACHO, CMS and CAP, and while maintaining the necessary state, federal, and insurance applications required to provide clinical diagnostic services.

## Data Analysis

For so many investigators, analysis represents the "black box" of the translational pipeline. It is reasonable to state that statistical analysis of the data is one of the most important steps in the translational process. In addition to determining the sample size and inclusion/exclusion criteria necessary to test a hypothesis, it is essential to utilize the correct tests of statistical significance during both discovery and application phases of the translational pipeline. This review will not detail all possible permutations of biostatistics, but we will touch briefly on certain statistical concepts. The average gene expression profiling experiment can generate in excess of 30,000 individual data points per clinical sample, while current high-throughput SNP-chips can generate >100,000 discrete attributes per analysis. Similarly, proteomic experiments generate vast amounts of coupled data that includes both fractionation (such as 2-D gels or 2-D liquid chromatography) and detection (such as mass spectrometry) elements. Coupled with extensive clinical content, this collectively presents significant challenges for data storage and retrieval, as well as statistical analysis. The most frequent frustration of new users of the "omics" technologies remains "What are the data telling me?"

Before proceeding with specific examples, it is important to grasp the concepts of normalization and data massaging or "filtering" [11]. To compare data across multiple samples, data is typically normalized or scaled such that results from one experiment are directly comparable to those of another. There are a number of methods to normalization across experiments including the use of a common reference sample or internal controls such as housekeeper genes/proteins that do not alter across experiments. Various forms of mathematical normalization can then be used (for example mean centering) to scale the results across experiments. As discussed above, through the tracking of protocols and minimizing experimental variation, one can reduce the degree of scaling required to directly compare data across different experiments. The normalization routines, as with pre-filtering of data, should be performed during an analysis session and not prior to data entry, since the different normalization and filtering routines can significantly effect the analytical results. As such, it is important to track the normalization and filtering criteria employed during analysis, such that results from sessions using different methods can be compared and contrasted.

**Figure 4:**
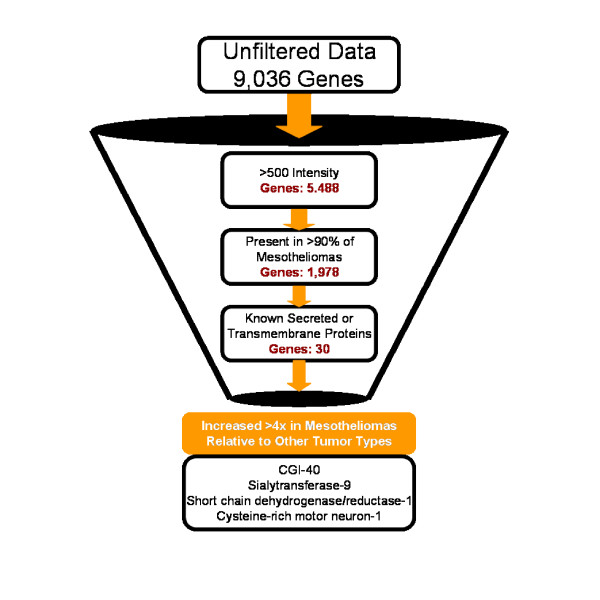
Sequential filtering of Affymetrix gene expression data to identify potential plasma biomarkers of mesothelioma. The key aspects of a disease biomarker include sensitivity and specificity. These can be partially addressed through logic-based filters within Dx2Tx.

One problem in dealing with vast amounts of clinical and/or multiplexed molecular data from a relatively small sample population is that many of the observed correlations could (not necessarily do) occur by random chance. One method investigators use to minimize the probability of this so called "false discovery" is to reduce the complexity of the data through successive rounds of filtering. Depending on the application, we do use some filtering under certain circumstances. However, as with normalization, this is done during data analysis and not prior to database entry. In this fashion, we can filter and analyze the data "on the fly", allowing the user to evaluate the effect of filtering the raw data. For example, if we were looking for a specific set of genes that are found highly expressed in mesothelioma relative to other tumor types, we may eliminate genes in mesothelioma samples below a certain expression (intensity) threshold, and those above a certain level in other tumor types. This would maximize the likelihood of identifying sensitive and specific mesothelioma genes, and minimize the gene pool and hence probability of false discovery. We also use fold-change filters depending on the application. For example, we may only be interested in diagnostic biomarkers that display at least a 4-fold increase in mesothelioma tumor tissue relative to other tumor types and normal mesothelium. In addition, genes with certain characteristics (such as those that encode only secreted or transmembrane proteins [12] can be filtered (included or excluded) within Dx2Tx. Thus, while it is important to note that these filters are not tests of statistical significance, they nonetheless can be used in the context of biological logic, to maximize the likelihood of identifying clinically valuable data within multiplexed data. The logic flow depicted in Figure 4 shows the effect of successive rounds of filtering the data from mesothelioma samples with the intent of identifying possible biomarkers that may be detected in blood or pleural effusions. This logic-based informatics approach to identifying possible disease biomarkers in physiological fluids can lead to candidate proteins that can be specifically detected in the corresponding blood/urine samples by other methods. We have shown the utility of this predictive approach in parallel with proteomic analysis of plasma in some experimental models of cancer. While this approach is yet to be validated in the human disease, we believe that this represents an excellent supplement to existing biomarker discovery programs that is readily implemented through simple data filters.

**Figure 5:**
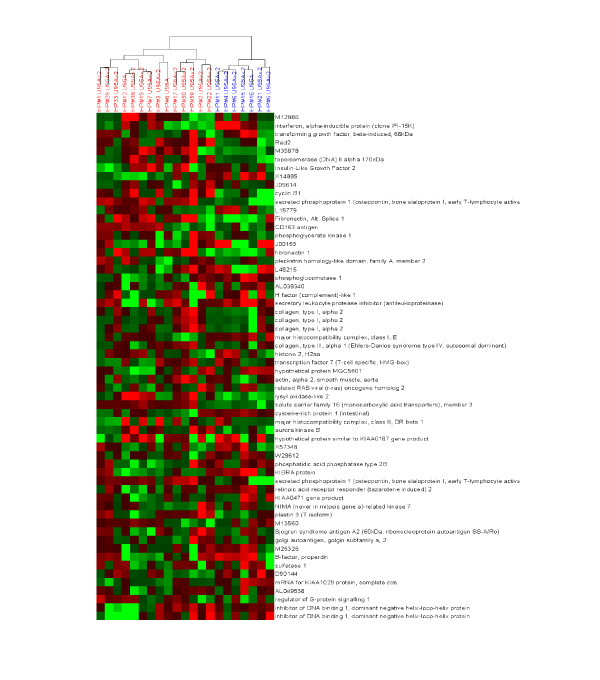
A two-color clustergram generated after hierarchical clustering of gene expression data (Affymetrix U95A) across 21 tumor samples collected from patients with mesothelioma. Clustering has been performed in only the X axis, such that samples are grouped based upon similarity in overall gene expression (the identified sample sub-groups are color coded). The gene expression data has been mean centered, such that degrees of red and green indicate relatively high and low expression of the corresponding gene respectively, while black represents the mean value across samples. In this fashion, the relative expression of many genes can be readily visualized across several samples simultaneously, and the relationships between samples observed.

**Figure 6:**
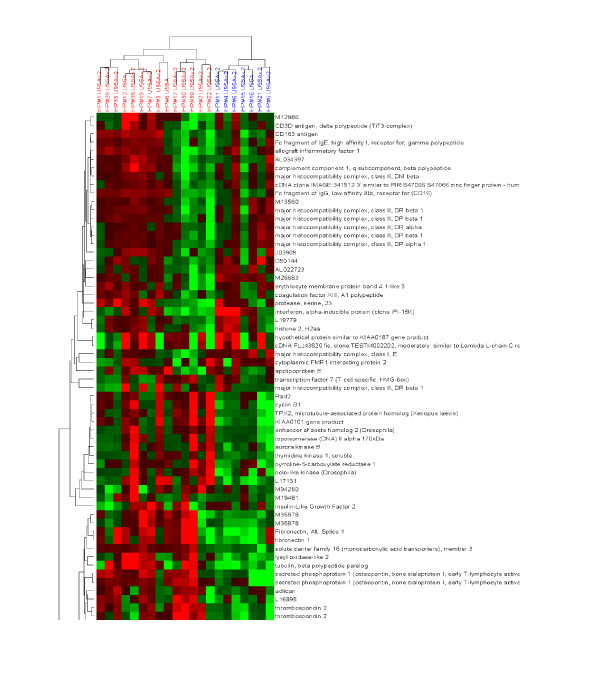
Hierarchical clustering of Affymetrix gene expression data as described in the legend to figure 5, with the exception that genes are also clustered based upon similarity in expression across the samples. In this fashion, correlations between samples and genes can be simultaneously observed.

**Figure 7:**
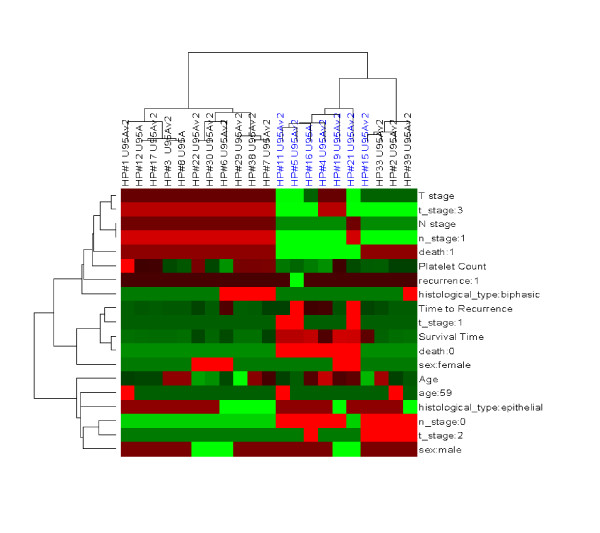
Clustering of mesothelioma tumor samples (X attribute) by clinical data (Y attribute) reveals possible epidemiological relationships between the various clinical features. Some well known relationships (such as stage of disease, lymph node status, death), as well as some less established patterns (such as a correlation between high platelet count and recurrence) are readily observed in this mode of operation. The degree of the clinical event is represented on a mean centered scale, such that red and green indicate relatively high and low extents respectively. A subset of samples has been selected based upon the extent of a single trait, in this case prolonged survival time (blue).

There are an abundant number of methods that can be employed during analysis of multiplex data to determine statistical significance. In the absence of becoming a quasi-expert in statistics, we highly recommend that the assistance of any number of statisticians is sort. The key is to understand why certain statistical tests are used under a variety of circumstances, and what the limitations of each test may be. We typically use similarity-based tests (such as hierarchical clustering, principle component analysis, multidimensional scaling, etc) to identify relationships between variables (clinical, experimental and molecular) within large datasets [13,14]. These metrics identify the degree of similarity (or difference) between various attributes, and are extremely powerful when attempting to discover inter-sample relationships based upon their molecular and/or clinical features. For example, unsupervised hierarchical clustering can be used to cluster the X attributes (such as mesothelioma samples) based upon the values of the Y-attributes (such as relative gene expression) as shown in Figure 5. In addition, the Y attributes can be clustered, based upon their similarity across the X attributes, providing a 2-dimensional clustergram displaying overall relationships (Figure 6). The co-clustering of samples is essentially a raw form of a molecular diagnostic application since samples with similar genotypes cluster based upon biological similarity (phenotypes). The co-clustering of genes and/or clinical data is also a potentially powerful application. For example, genes/proteins with similar functionality are often co-regulated at the level of their expression, and hence typically "co-cluster" on a gene expression clustergram. This concept becomes particularly powerful when attempting to predict the function of unknown genes based upon their overall correlation with a gene of known functionality [15-17]. In the case of clinical data, inter-relating clinical and/or environmental events can also be performed. Hence, features that correlate (such as increased stage of disease and poor outcome) are typically adjacent on the clustergram (Figure 7). Coupled with the extensive collection of standardized clinical data highlighted throughout this review, this feature alone may have significant impact in the mining of clinical data in disciplines such as epidemiology.

**Figure 8:**
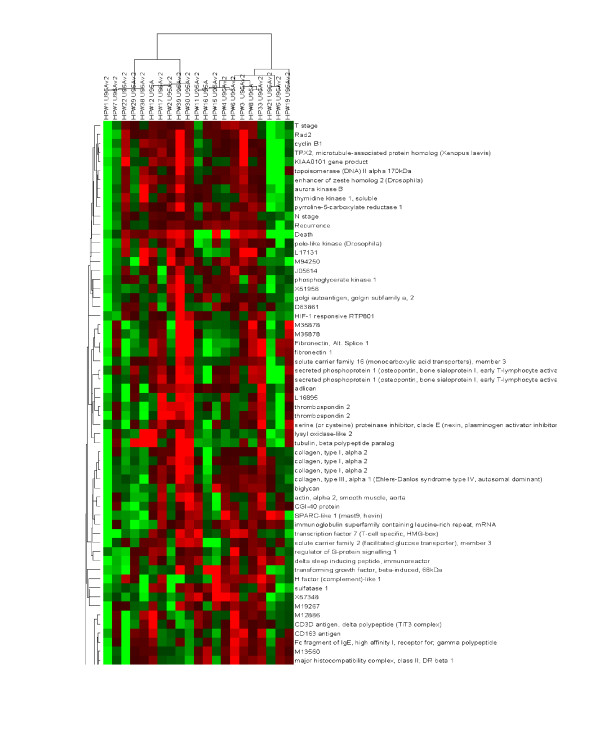
Clustering of samples based upon integrated clinical, experimental and molecular attributes. In this sense, molecular-clinical (genotype-phenotype) associations can be readily observed. As described in the legend to Figure 7, the extent of the clinical/molecular attribute is represented on the same normalized scale, such that red and green represent relatively high and low values respectively.

**Figure 9:**
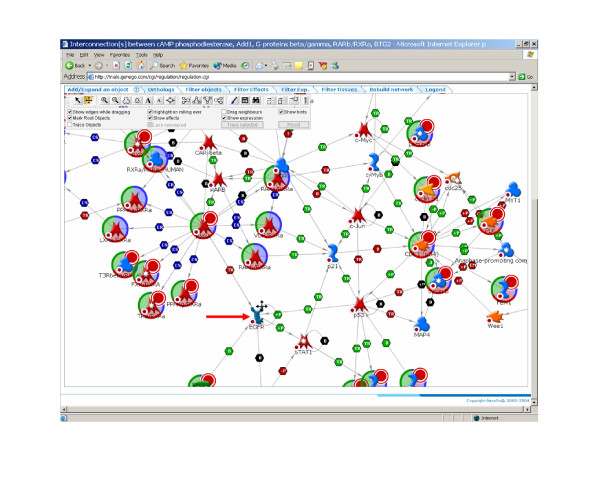
Mapping molecular correlates of aggressive mesothelioma to highly curated molecular pathways can identify the underlying molecular mechanisms of the disease. This information could be used for diagnosis, as well identification of the key steps that may represent intervention points in the treatment of the disease. The red arrow indicates a predicted therapeutic target (EGFR). Pathway mapping was generated using MetaCore™ (GeneGo, Inc., St. Joseph, MI). For more information on this pathway mapping tool, see .

In addition to correlating clinical and molecular data individually, these data types can be merged and viewed simultaneously within the same clustergram. In this fashion, possible associations between clinical, experimental and molecular features can be readily identified. For example, as shown in Figure 8, there appears to be an association between T-stage and a number of genes known to be involved regulation of the cell-cycle. Classification of gene/protein function (so called "annotation") can provide important information with regards to the underlying molecular cause(s) of a clinical event. In addition to utilizing the publicly available annotations (for example see Gene Ontology ), we also map results to molecular pathways using detailed pathway mapping software now available. Taking this approach, the coordinated expression of genes/proteins can be seen to map to specific molecular networks, therefore providing important information as to which pathways maybe activated or in-activated in association with a clinical event. For example, when all the genes differentially expressed in recurrent mesotheliomas relative to non-recurring tumors at a defined statistical significance (p < 0.001) are mapped using the MetaCore™ software (MetaCore™ ), a clear signaling pathway associated with cell proliferation is identified that appears to be hyper-activated in aggressive mesothelioma tumors (Figure 9). As discussed below, in addition to providing clear diagnostic value, this information is particularly useful in the design of treatment strategies that may target key points within the identified molecular network.

## Clinical Diagnostics

With respect to identifying patterns (hypotheses) within the complex clinical and molecular datasets that could be translated into clinical diagnostic applications, we typically begin with unsupervised clustering techniques such as hierarchical clustering as shown above in Figures 5, 6, 7. In this fashion, sample similarity with respect to clinical, experimental and/or molecular attributes can be assessed. Dx2Tx extends these analyses to identify clinical and/or experimental variables that statistically correlate with defined sample sub-groups. During this step of hypothesis generation, Dx2Tx runs back into the database housing all of the standardized clinical and experimental data and identifies correlates of the selected sub-groups. This is a highly powerful utility when operating in unsupervised mode, and requires an intricate link between data analysis and database content. Unsupervised clustering may for example identify the degree of molecular similarity across a cohort of patient samples, which could identify several clearly delineated groups at the genotype level. Running in hypothesis generation mode, Dx2Tx then identifies statistically significant correlates of these groups, and assigns clinical/experimental features to each. When Hypothesis Generator was executed on the 2 sample subgroups highlighted in Figure 5, the clinical features time to recurrence (p = 0.003), T-stage (p = 0.004), survival time (p = 0.0002) and platelet count (p = 0.005) were returned as significant correlates of these sub-groups. Thus, while these samples may have been initially collected in the context of a different user-defined hypothesis, through the collection of standardized clinical data in addition to the generation of multiplexed molecular data, Dx2Tx was able to identify statistically significant patterns (hypotheses) within the data in an unbiased fashion. The user can of course decide which hypothesis to pursue. In this example, our ability to potentially utilize gene expression profiling to predict survival time of mesothelioma patients following surgery based upon gene expression within the tumor would have obvious prognostic value. Therefore, the next step would be to test this hypothesis and determine the accuracy of a possible diagnostic test.

Once a hypothesis has been generated, Dx2Tx identifies samples against which the hypothesis can be tested. Certain inclusion and exclusion eligibility criteria can be considered and used to filter the content of the database to identify subjects/samples/experiments with certain characteristics. Dx2Tx also allows samples to be selected based upon the extent of any attribute(s) (Figure 7). For example, the investigator may be primarily interested in only a subset of the sample population that displayed the greatest and least extensive toxicity to a given drug. This is assisted through the selection of the trait of interest and setting the extent of the trait (i.e. by defining standard deviations from the population mean). This feature may be particularly important in retrospective analysis of large clinical trial cohorts, since the outliers for a given trait can be identified prior to sample procurement.

Once the sample population is selected, we test the hypothesis across the series of selected samples in a 2-step process. A subset of the samples (typically defined as a training set) are selected (either logically or at random) from each subgroup (for example disease versus control) to develop a discrimination algorithm that identifies statistical correlates of the feature in question. It is worthwhile to note that Dx2Tx identifies clinical, experimental and molecular correlates of the selected feature(s), thereby integrating both clinical and molecular data into the potential diagnostic algorithm. The user can exclude any attribute from the input to the training algorithm. In a second cross-validation test, the trained algorithm is applied to the remainder of the samples (in retrospective mode of operation, with known outcome), to determine if the test could have accurately predicted the nature of the remaining samples. The outcome of the test is plotted using a receiver operator characteristic (ROC) curve to determine the accuracy of the test. The ROC curve is a way to visualize and quantify the effectiveness of a procedure by graphing the true positive rate (Y axis) against the false positive rate (X-axis). The area under the curve (AUC) provides an approximation of the accuracy of the test. A procedure with no effectiveness (AUC = 50%) would show a random 1:1 line, indicating that for every true positive, the procedure also generated a false positive. Generally, an AUC of 90–100% is considered excellent, while an AUC of 80–90% is good. The ideal diagnostic test would of course identify all true positives before encountering a false positive (AUC = 100%).

**Figure 10:**
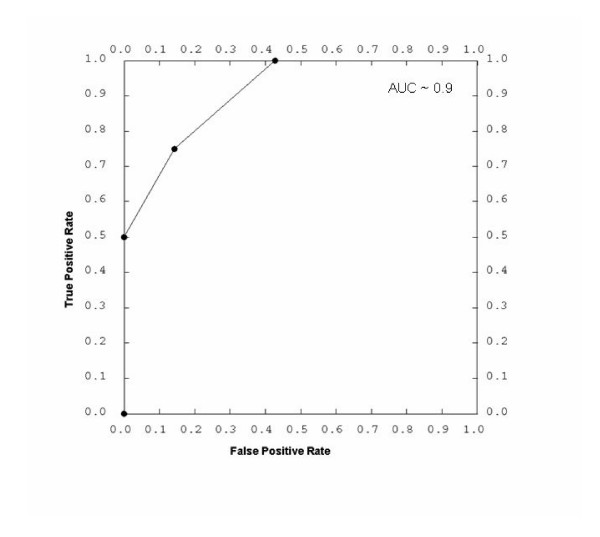
A Receiver Operating Characteristic (ROC) curve showing the performance of an integrated clinical and molecular diagnostic test for predicting prognosis of patients with mesothelioma. The Area under the curve (indicative of the tests accuracy) is approximately 90%.

In this working example, the hypothesis generated from analysis of unsupervised clustering of gene expression data from mesothelioma tumors is that survival time of patients can be predicted based upon the underlying genomic signatures of the tumor. Thus, patients with the shortest and longest survival time following surgery were placed into two groups. Each group was then randomly divided into 2 additional groups, the training set and the test set. The discriminating clinical and molecular features are first identified using a standard t-statistic for numerical data and chi squared for binary (including text) data. This test statistic is then used in a weighted voting metric [18]. Data are first converted to a respective z score in order to normalize data of different types to a similar scale. A more refined statistical package, which will more rigorously integrate the binary and non-binary data, is currently in the process of being implemented into the Dx2Tx solution. In this fashion, the experimental, molecular and clinical attributes that statistically correlate with survival time are first identified. In this example, no experimental variables (i.e. those which may denote a variation in experimental protocol or quality) were identified that correlated with patient survival time. The clinical parameters platelet count and T-stage were identified as clinical correlates of survival time and therefore included into the training algorithm. In addition, 157 genes were identified, the expression of which correlated with survival time (p < 0.05). Each attribute (platelet count, stage, and the 157 individual genes) was then weighted based upon the calculated t-statistic within the training group. A discrimination score (the sum of the t-statistic multiplied by the normalized z-score for each attribute) was then calculated for each sample within the training groups and a threshold decision point (the value at which a sample is classified as neither group 1 or 2) is set halfway between the means of the two test groups. Alternatively, a user can set the threshold in order to maximize either sensitivity or specificity of the assay, or set it to a value which would demarcate an acceptable test failure rate. In this fashion, the end-user can set the decision point of the classification algorithm on the side of false positives or false negatives based upon the clinical consequence of the test result. For example, if a positive test results in administration of a poorly tolerated treatment, the physician would typically error on the side of false negatives. At this time, a discrimination score is calculated for the remaining test samples, compared to the threshold decision point, and assigned a classification. The predicted classification is then compared to the actual outcome. While complicated, Dx2Tx performs this cross-validation metric in a matter of seconds. In this working example, the ROC plot generated from the prediction of the prognosis of patients with mesothelioma suggests that this particular diagnostic test is approximately 90% accurate at determining the 6 month survival of patients following surgery as determined by the area under the ROC curve (Figure 10). Once validated, the classification algorithm is stored within Dx2Tx, such that it can be applied to any future sample. Thus, through the capture of standardized clinical, experimental and molecular data, hypotheses can be rapidly generated and tested, and further developed into potentially useful diagnostic applications. At this point, the focus may shift from retrospective analysis to prospective studies.

## From Diagnosis to Treatment

In addition to the identification of potential diagnostic applications, we have a major focus on identifying new treatment targets, and/or improving therapeutic strategies involving existing treatments. Patient variation, with respect to treatment response (efficacy and toxicity), is a well documented phenomenon [1]. Through the capturing of clinical data and pertinent samples across a large patient population that exhibits variable treatment response, retrospective statistical analysis of the integrated clinical, experimental and molecular data could reveal the underlying causes of this variation. For example, DNA polymorphisms in some isoforms of the cytochrome p450 enzymes have been associated with the variation in the rates of metabolism of many pharmaceutical drugs across a sample population [19]. As a result, a specific test now exists that could be used to better determine the optimal dose of some pharmaceutical treatments (Roche Release of AmpliChip ). These so called "companion diagnostics", which could accompany therapeutic agents and assist in treatment decisions, are also being developed for specific agents that display varying degrees of efficacy and toxicity across sample populations. With the accurate capture of longitudinal clinical data including toxicity and response assessment, associations between clinical response and molecular features of either the patient and/or the disease tissue should be readily identifiable within complex retrospective datasets. For example, we have identified a genomic signature within plasma cells isolated from multiple myeloma patients that correlates with tumor response to the drug melphalan. This genomic signature is currently being applied to additional patient samples using cross-validation statistics as outlined above, to determine the accuracy of this possible companion diagnostic application. As with most single agent treatment regimens, drug resistance in the area of oncology represents a significant problem in the treatment of the disease. Therefore, pre-treatment tests could conceivably identify the patients who would benefit the most and least from treatment.

Our research also includes the discovery of possible early surrogate markers of therapeutic index. This ideally requires the collection of clinical specimens and data both pre and post treatment. In conjunction with the treatment of cell lines in culture and molecular analysis of livers and kidneys from treated mice, early biomarkers of efficacy and toxicity have been identified in association with several treatment regimens. If these biomarkers of response can be validated in retrospective patients, they could be written into future clinical studies to provide an early indication of therapeutic effect. These biomarkers may ultimately be used as surrogate markers to determine discontinuation or modification of protocols to maximize therapeutic index in prospective trials.

There are currently a number of drugs in development, in clinical trials or that have recently received FDA approval that target specific molecular aberrations [20-23]. Unlike cytotoxic chemotherapies, molecularly targeted therapeutics often display a high degree of specificity against the selected target. Based upon the specificity of these drugs to defined proteins, it is envisioned that molecular-based diagnostics will naturally accompany these agents to identify the patient sub-population who will benefit from treatment. Because of the inherent genomic instability of cancer, combinations of these molecularly targeted drugs will almost certainly be required to ultimately treat the disease. Indeed, mathematical models of adaptive microevolution of the cancer cell suggest that a multi-modality treatment strategy that targets at least five individual molecular targets simultaneously will be required to minimize the chance of a single cell within the tumor acquiring resistance to each agent [24]. As part of our research effort, we are attempting to identify the optimal multi-modality targeting strategies to treat specific tumor types based upon their molecular makeup. For these reasons, we have incorporated a drug-target database that is regularly updated to include molecularly targeted agents as they are publicly disclosed. During analysis, we can filter the datasets to only include genes/proteins against which drugs have already been developed. For example, we can substitute the drug-target list as a filter in place of the known secreted or plasma membrane proteins discussed in relation to Figure 4. When performing this function on the mesothelioma dataset, epidermal growth factor receptor (EGFR) inhibitors in combination with eniluracil and topoisomerase II inhibitors are identified as a possible combination treatment for mesothelioma based upon relatively high expression of their molecular targets EGFR, Dihydropyrimidine dehydrogenase and topoisomerase II respectively. Such hypotheses obviously require testing in a relevant preclinical model of the disease (see below). Nonetheless, because the corresponding drugs have already been developed, this is a readily testable hypothesis assuming the investigator can gain access to the therapeutic agent. As discussed below, however, we do not believe that expression levels of the molecular target alone are necessarily sufficient to predict drug efficacy.

The FDA approval process of the EGFR inhibitor Iressa has received a great deal of attention [25]. Recently, it was shown that lung carcinomas from a subset of patients that possess activating mutations in the EGFR are most responsive to Iressa [26,27]. This raises an important concept we have been pursuing for some time; namely that diagnostic tests need to address activity of the target(s) rather than merely expression levels. Since an active molecule can often shut down its own expression, while conversely, hypo-active targets may consequently be over-expressed, this biological phenomenon of negative feedback often results in inverse expression-activity relationships (CPW, unpublished observations). Hence, in combination with gene silencing technologies (such as RNA interference) that mimic a selective drugs action, we are identifying the down-stream genomic and proteomic consequences of target gene disruption for the purpose of identifying biomarkers that could be used to assess target activity. We believe that rather than using expression levels of the target molecule alone as a rudimentary diagnostic test, this more sophisticated approach may yield greater success in attempting to predict the optimal treatment strategies based upon a molecular profile.

In addition to pursuing existing drug targets, we are also attempting to identify novel targets that may warrant future drug discovery efforts. We typically attempt to validate only candidate genes/proteins that have or are predicted to have "drugable" characteristics. To determine whether a potential target is drugable, we are currently using some relatively simple criteria based upon the classes of drug targets that have been actively pursued by pharmaceutical/biotech companies to date. For example, we have annotated the publicly available drug targets described above using gene ontology and literature mining tools within Dx2Tx [9], and identified several recurring features of these targets (such as kinases, phosphatases, G-protein coupled receptors, etc). Any gene/protein identified with the same annotation is "drugable" by simple association. We also identify genes/proteins that co-cluster with genes/proteins with drugable features, since as described above co-expressed genes/proteins often share similar functionality. Future developments will include sequence, domain and structural-based predictions of drugable characteristics, but it remains to be seen if this selective approach will lead to accelerated clinical application in the future. Nonetheless, using the combined content and analytical power of Dx2Tx we can sequentially filter data in an attempt to identify specific targets for the disease in question and condense our target candidates further to identify those with the greatest potential for drug development.

## Preclinical Models of Disease

The majority of the preceding discussion has focused on the identification of molecular correlates of disease, and identifying those that may represent diagnostic biomarkers and/or treatment targets for intervention. A large proportion of our translational research effort is dedicated to functional validation, where through various means, the expression and/or activity of the target are modified and the functional consequences addressed. While determining the function of a diagnostic biomarker is not necessary, the functional consequence of target gene/protein disruption is essential when establishing the true therapeutic value of a potential target. An ideal therapeutic target would be one that is causative of the disease, and inhibitors against which thus cause disease regression. The following discussion will focus on a high-throughput means by which we assess the functional significance of target gene/protein disruption in murine models of various human malignancies.

There are a variety of approaches one can take to interfere with gene/protein expression and/or function with the purpose of demonstrating a definitive role in a biological process [28]. These include the use of pharmacological agents, antibodies and/or interfering mutants. However, these approaches require reagent access and/or some in-depth knowledge about the gene/protein in question. These approaches are also relatively low throughput and expensive, and can result in a significant bottleneck effect as potential targets are identified during the translational research effort. Antisense technologies represent an excellent approach to determine gene function, and are readily integrated into gene/protein discovery programs due to the wealth of gene/protein sequence information now available [29,30]. In this regard, the field of RNA interference has emerged as a highly specific and relatively simple way to disrupt genes [31]. RNA interference (RNAi) is a conserved phenomenon, whereby long double stranded RNA (dsRNA) is processed into small 21–23 nucleotide dsRNA fragments, termed short interfering RNA (siRNA) [32]. These fragments then target and degrade highly homologous RNA gene transcripts, thereby inhibiting gene expression in a sequence-specific manner. RNAi is thought to function to maintain genomic stability, regulate cellular gene expression, and defend cells against viral infection [33,34].

We have developed an avian retroviral vector that can deliver siRNA to cells expressing the viral receptor (TVA) [35]. TVA expression can be directed to specific cells in vitro through exogenous transfection/infection, or in vivo through transgenic technology [36]. This system was developed to allow us to target the delivery of gene-specific siRNA to tumor and/or endothelial cells in vivo, such that the effect that target gene disruption has on tumor growth, metastasis and angiogenesis can be directly assessed. As discussed above, we typically target genes/proteins that we predict to have "drugable" characteristics, and in a sense this retroviral-siRNA approach mimics the optimal molecularly-targeted drug due to its targeted delivery and exceptional degree of gene specificity. In addition, these avian retroviruses do not replicate within mammalian cells and as such cells can be infected multiple times with vectors targeting multiple genes. This system therefore allows us to investigate the functional consequences of combinational targeting strategies. Because of the particular cloning strategies we have incorporated, it takes only six weeks from the discovery of a potential target gene to the point of assessing functional consequences of gene disruption in a relevant preclinical model of the disease. Because of the efficiency of the system, we are able to assess multiple targets simultaneously. For example, we are currently evaluating 19 targets in various combinations that have been predicted to display optimal efficacy in murine models of mesothelioma, pancreatic cancer, colorectal cancer and multiple myeloma. Thus, by introducing a systematic approach to target validation, we have limited the bottleneck between target discovery and functional validation.

Improved murine models that depict specific features of the human disease in question are essential to validate the *in vivo *significance of experimental findings in a relevant preclinical setting. We particularly focus on the development of orthotopic xenograft models, in which human tumor cells are implanted into their corresponding site of origin within an immune compromised mouse. In this fashion, human tumors form in the tissue site of origin that more closely resemble the human counterpart with respect to biological behavior. These orthotopic models better recapitulate the various stages of tumor progression and more accurately reflect responsiveness to various therapeutic intervention strategies [37]. Coupled with *in vitro *and *in vivo *delivery of siRNA against multiple target genes, these preclinical models readily allow us to evaluate the requirement of proposed target genes in tumor progression. Through these studies, we are beginning to identify the optimal combination targeting strategies that may lead to the eventual treatment of aggressive cancers.

## Moving from Retrospective Analysis to Prospective Clinical Trials

In the vast majority of cases in which molecular data is being correlated with clinical events, a hypothesis-driven inquiry is tested against archived clinically documented specimens. The majority of these trials are asking either a classification question (i.e. what profile sets this tumor apart from other tumors), a prognostication pattern (what group of genomic/proteomic patterns will predict time to progression or time to death), early detection (how is this tumor different from its "cell of origin" at the earliest time point recognizable such that a genomic/proteomic pattern could predict the development of malignancy in a high risk population), or response to therapy (is there a de novo set of genomic/proteomic parameters which predict either response or resistance to a given therapy). For classification phenomena, the investigator must currently rely on pathologic differences to stratify tumors into clusters which, upon genomic/proteomic analysis, have consistent and congruent distinguishing features. This is probably the easiest of analyses and relies mainly on established architectural and morphologic differences between tumors instead of clinical behavior and endpoints. Nevertheless, as with all discovery test-sets, validation prospectively must be performed in order to test the accuracy of the classification algorithms. This requires prospective application of the algorithm to blinded samples, and comparing the predicted outcome with the actual observed pathology.

For the early detection, prognostication, and prediction of therapeutic response, one must link potential diagnostic tests to newly developing trials to allow validation of the established hypothesis. Before undertaking such an effort, however, the investigator must realize that the data were typically derived from a set of patients within a particular treatment regimen. For example, the patients presented in this article were characterized as having specimen procurement prior to definitive cytoreductive surgery followed by adjuvant therapy, and therefore a prospective trial which conforms to this treatment regimen should ideally be designed to avoid confounding variables. For the validation of diagnostics that predict time to recurrence or survival following treatment, a phase II trial of surgery and/or postoperative adjuvant therapy could be designed in which samples are harvested at the time of surgery. The diagnostic test would then be performed on the clinical specimen to obtain a prediction of patient outcome, and the patients' clinical course followed to see if there is prospective validation of the test. These analyses, however, must be performed in patient groups not weighted towards either high or low risk patients, and indeed, some stratification of clinical parameters should be specified at the outset in order to make reasonable comparisons. This is especially true if the molecular data is to be validated as part of a Phase III randomized trial comparing two treatment regimens after surgery. As the content of Dx2Tx expands to include clinical trial data, analysis can be performed on only those patients that conform to specified clinical parameters. Therefore, with the complete and standardized collection of clinical data from a large population of patients receiving various treatment regimens both on and off of protocol, established hypotheses can be further tested on additional retrospective subjects; in a sense a virtual trial that begins to more closely resemble the carefully controlled prospective clinical trial.

The ideal clinical trials addressing the validity of molecular data to predict a clinical parameter are those involving patients with no prior diagnosis or treatments, who subsequently receive a single therapeutic regimen. Preferably, both pre- and post-treatment specimens are obtained from these patients. Such a pure trial could, for example, test whether a predefined genomic/proteomic pattern could predict a poor outcome despite favorable clinical parameters. Trials of patients receiving initial chemotherapy could also help to define the fidelity of the omics technologies for the prediction of chemosensitivity. These hypotheses may have been derived from a retrospective set of patients receiving the same chemotherapy or targeted therapy for which outcomes were known, or from the *in vitro *discovery of genomic/proteomic patterns defined in cell lines treated with the corresponding agent(s).

Early detection clinical trials using genomic and/or proteomic technologies will typically take the longest to validate. After the discovery of proteins as candidate markers from analyses of serum, urine, or other fluids in patients with early stage malignancies compared to the appropriate high risk group, cohorts of high risk individuals (e.g. asbestos exposed or tobacco smokers) must be identified who are willing to have collections of the appropriate specimens longitudinally at given intervals. Moreover, in initial trials of these markers, the investigator must define at what time to disclose the value of the measured biomarker. One possibility would be to allow the prospective clinical trial to reach a certain endpoint (i.e. a certain number of cancers occur in the cohort which are detected by the standard of care radiographic or physical examination) and then reveal the results of the marker to see whether there is predictive value. Another way would be to define the value of the marker at follow-up intervals during the course of the trial and, in the face of radiographic or physical findings, initiate an invasive workup to "find" the predicted cancer. It is generally agreed that, although a two stage validation effort takes longer (i.e. blinded values for the marker in question until completion of the study followed by a study with invasive or semi-invasive investigations based on fluctuations or absolute levels of the marker), such a model is currently preferred.

With respect to novel treatment strategies predicted from retrospective analysis and/or through the preclinical studies defined above, the next logical step is to attempt to validate the retrospective data which initially pointed the investigators to these potential treatment options. If the new therapy has never been used in humans before, it is important to define the maximum tolerated dose of the treatment. This is defined as the dose at which, in a Phase I clinical trial, a defined fraction (typically one third) of the patient cohort develop dose limiting toxicity. Once the dose or treatment strategy is found to be acceptable, a Phase II trial is designed to provide a measure of the activity of the agent(s) and to begin to define whether there is any benefit of the regimen in certain patient cohorts. The use of tools such as Dx2Tx could be invaluable in the future tracking and analysis of such trials, especially if samples are prospectively harvested at various times pre and post treatment. For example, in the Phase I/II trial, genomic/proteomic correlates of clinical toxicity could be readily identified by melding the clinical data with "omic" data from the patients who have undue toxicity. Molecular pathways that intuitively result in toxicity could be identified which could possibly be abrogated with another agent. With respect to determination of therapeutic efficacy, our present means of defining "clinical correlates of response" is essentially a "best guess" or subjective prediction of what clinical markers may indicate that the new agent is yielding a therapeutic effect. A more objective measure of downstream events resulting from an efficacious agent would result from analysis of the molecular data in tandem with clinical information for the responders compared to the non-responders. Dx2Tx would be able to then define the most important markers of response, both clinical and molecular, which could then be validated in a Phase III trial assessing agent efficacy.

## End Use

It is unknown when an integrated clinical/molecular evaluation of the suspected or afflicted cancer patient will be of use to the end user, the practicing physician. Certainly, many of the same arguments that are used for and against "genetic testing" in other diseases may be used for such a global approach to oncology. There is no doubt however, the ability to define clinical behavior in a more efficacious and predictive manner, other than the archaic prognostic indicators which we use today, will help clinicians initiate and/or alter treatment course earlier and guide clinicians toward informed discussions with their patients in order to make treatment and/or surveillance decisions. If indeed the fidelity of combinatorial molecular and clinical medicine proves to be satisfactory, we may then be able to spare patients unnecessary treatment interventions which are currently doomed to failure. Moreover, the medical oncologist will be able to choose the correct cytotoxic and/or targeted therapy based on a global clinical-molecular snap-shot, which should translate into more favorable health economic policies and patient outcomes.

## Conclusions

Throughout this review, we have highlighted the importance of designing research protocols involving human subjects that permit the collection of not only clinical specimens, but also extensive standardized clinical data in a longitudinal fashion. Through the merging of clinical and molecular data, non-biased patterns can be discovered that could translate into novel diagnostic and/or treatment opportunities. We have introduced a methodical approach for archiving and mining these seemingly disparate data sources that we believe can accelerate the translational research discovery pipeline. While there are clearly improvements to be made in the systematic collection of accurate and nationally standardized clinical data, we believe the integration of medical informatics and the molecular technologies can convert the once visionary concept of molecular-based medicine into a present reality.

## Competing Interests

CW – A provisional patent has been filed on the Dx2Tx system described in this article.

HP – This author declares that he has no competing interests.
